# Acute Decrease in Glenohumeral Internal Rotation During Repetitive Baseball Pitching Is Associated with Transient Structural Changes in Medial Longitudinal Arch of Stride Leg: Pilot Study Using Mixed Model

**DOI:** 10.3390/sports13120446

**Published:** 2025-12-10

**Authors:** Takeru Abekura, Noriaki Maeda, Tsubasa Tashiro, Satoshi Arima, Ryosuke Kaizuka, Madoka Koyanagi, Koshi Iwata, Haruka Yoshida, Ginji Ito, Mayu Ueda, Takashi Yamada

**Affiliations:** Graduate School of Biomedical and Health Sciences, Hiroshima University, Hiroshima 734-8553, Japan; tabekura1124@gmail.com (T.A.);

**Keywords:** foot/physiology, glenohumeral internal rotation deficit, ground reaction force, intrinsic foot muscles, shoulder joint/physiology, throwing

## Abstract

Pitching requires effective transfer of ground reaction force (GRF), and structural breakdown of the medial longitudinal arch (MLA) may influence glenohumeral internal rotation (IR) deficits. This study investigated whether changes in foot morphology of the stride leg and soft tissue characteristics are associated with loss of IR during repeated pitching. Fifteen male college pitchers completed 60 pitches in a simulated game. IR range of motion (IRROM) was assessed before and after pitching. The navicular height, mechanical properties of the abductor hallucis (AbH) and plantar fascia, and GRF were measured at multiple time points. Correlation analysis and a linear mixed model were used to identify predictors of IRROM change. The mean change in shoulder IRROM during pitching was −21.9° ± 8.4°. IRROM and navicular height decreased significantly over time. The AbH elasticity increased throughout the pitching sequence. Greater reductions in IRROM appeared related to a higher vertical GRF (*p* = 0.021) and increased AbH elasticity (*p* = 0.046). Vertical GRF was unrelated to fastball velocity (*p* = 0.260), whereas anteroposterior GRF correlated with fastball velocity (*p* = 0.038). Morphological and mechanical changes in the stride leg, particularly within the support of the MLA, can influence IRROM. Reducing vertical GRF and stress on the AbH may help preserve the IRROM without compromising performance.

## 1. Introduction

The glenohumeral internal rotation deficit (GIRD) is a well-recognized adaptive phenomenon in overhead-throwing athletes, characterized by reduced internal rotation range of motion (IRROM) in the dominant shoulder. Clinically, GIRD has classically been defined as a reduction of more than 20° in internal rotation range of motion (IRROM) of the dominant shoulder compared with the non-dominant side [1]. In recent years, the need to evaluate GIRD in conjunction with total rotational motion has been increasingly emphasized [2]. In the field of baseball throwing injuries, shoulder rotational range of motion is an important factor for injury prevention, and GIRD has been suggested by meta-analyses to be strongly associated with throwing-related shoulder injuries [3]. From a kinematic perspective, recent studies have further investigated the mechanisms underlying this relationship. GIRD is associated with decreased posterior tilt of the scapula and increased external rotation at the glenohumeral joint, which may induce internal impingement and increase the risk of labral and rotator cuff injuries during pitching [4].

The mechanisms of GIRD differ between its chronic and acute forms. In adults, GIRD typically develops gradually due to thickening (morphological changes identifiable in images) and contracture (changes in mechanical properties such as tissue stiffness and extensibility) of the posterior capsule, with a moderate correlation (r = 0.40) reported between posterior capsule thickness and GIRD [5]. Conversely, in acute cases, transient reductions in IRROM may occur after pitching. This is caused by increased tension in the posterior shoulder structures, including the posterior capsule, infraspinatus, and teres minor, which can persist for up to 4 days [6]. These findings indicate that amateur baseball pitchers may throw while experiencing acute GIRD, emphasizing the importance of identifying and addressing factors contributing to acute increases in GIRD as part of injury-prevention strategies. Notably, thickening of the posterior capsule, which is one of the key structural components observed in chronic GIRD, is considered to result from the accumulation of microtrauma induced by repetitive throwing [7]. Athletes who exhibit greater transient GIRD during repeated pitching may be more likely to sustain substantial microdamage to the posterior capsule with each throw. This implies that investigating the underlying factors contributing to transient GIRD is not only important for short-term shoulder health but may also play a critical role in preventing the long-term structural adaptations associated with chronic GIRD.

Although throwing injuries are frequently studied as localized shoulder injuries, the development and progression of GIRD may be influenced by the entire kinetic chain. Pitching involves the sequential transmission of force from the lower extremities, through the trunk, to the upper extremities [8,9,10]. Disruptions along this chain can alter biomechanics and increase mechanical stress on the shoulder joint [11,12]. During the acceleration phase of pitching, when shoulder torque peaks within just 0.03 to 0.04 s, the stride leg plays a crucial role in stabilizing the body and absorbing the ground reaction force (GRF), which can reach up to twice the athlete’s body weight [13,14]. The direction and magnitude of this force influence performance metrics, including fastball velocity [15], and the horizontal braking component of the GRF strongly correlates with trunk-to-arm energy transfer (r = 0.68–0.72) derived from joint moments [16]. Furthermore, reduced dynamic balance of the stride leg—particularly in the posterolateral direction of the Y-balance test, reflecting the lower-limb position during the arm cocking and acceleration phases of pitching—has been identified as an independent risk factor for shoulder pain in high school pitchers, with a 5.8% increase in odds for each unit decrease in score [17]. These findings highlight that insufficient stride leg stability may impair kinetic chain efficiency, increase mechanical load on the shoulder, and potentially contribute to GIRD progression.

The medial longitudinal arch (MLA) plays a critical role in shock absorption and efficient postural control during dynamic movement. Postural abnormalities of the MLA, including flatfoot and high arches, have been associated with a greater history of shoulder or elbow surgery among elite baseball pitchers [18], suggesting that the structural integrity of the arch is clinically relevant in preventing throwing-related injuries. The MLA is supported by active components, such as the intrinsic foot muscles, with the abductor hallucis (AbH) playing a particularly important role in dynamic arch support, as well as passive structures, including the plantar fascia (PF) [19]. The increased stiffness of the AbH has been linked to improved postural stability during high-load tasks such as single-leg landings [20], involved in maintaining foot mechanics during pitching. In baseball pitching, the stride leg provides a stable base of support, facilitating efficient rotation of the pelvis, trunk, and throwing arm [21]. Flattening of the MLA has been associated with increased rearfoot and midfoot eversion during weight-bearing tasks, which reduces passive foot stability and may hinder effective lower-limb power generation during dynamic movements [22]. This mechanism suggests that MLA flattening may compromise the ability of the stride leg to function as a stable support base, resulting in less efficient rotation of the pelvis, trunk, and throwing arm, and potentially inducing compensatory pitching mechanics in which energy lost in the lower limb must be offset by the upper limb.

Interestingly, although the foot structure is often evaluated statically, recent studies have suggested that it can change dynamically in response to repetitive loading. A previous study found that navicular height decreased after a 10 km run, likely reflecting functional changes in the intrinsic foot musculature [23,24]. Furthermore, the flattening of the MLA may lead to elongation of the AbH, which in turn could contribute to increased muscle stiffness in flattened feet [25]. These findings suggest that the MLA is not a rigidly fixed structure, but rather one that may collapse rapidly under repeated mechanical stress, potentially accompanied by changes in the mechanical properties of the muscles involved in supporting the MLA. This transient breakdown may also occur during the acceleration phase of pitching, when the GRF peaks. This sudden arch collapse can disrupt force transmission and postural control, increase the mechanical stress on the shoulder, and potentially accelerate the progression of GIRD.

However, no studies have directly examined whether such acute morphological changes in the foot occur during repeated pitching or how they might influence shoulder function. Understanding this relationship could inform new conditioning and injury prevention strategies that integrate foot structure into shoulder health management for pitchers.

Therefore, this study aimed to descriptively clarify how navicular height and the mechanical properties of the AbH and PF in the stride leg change during repeated pitching, and to explore how these changes are associated with GRF and the decrease in IRROM. In addition, we sought to hypothesize the potential mechanisms that may underlie the relationship between pitching-related injury risk and foot morphology [18]. We hypothesize the following:

1. Repetitive pitching would acutely decrease shoulder internal rotation range of motion (IRROM).
2. The acute decrease in IRROM would be associated with changes in navicular height and the mechanical properties of the AbH and PF on the stride-leg side.

## 2. Materials and Methods

### 2.1. Sample Size Determination

An a priori power analysis was conducted using G*Power 3.1.9.2 (Heinrich Heine University Düsseldorf, Düsseldorf, Germany) (F tests: analysis of variance [ANOVA]: repeated measures, within factors) to determine the minimum sample size required to detect within-subject changes in shoulder IRROM across four timed measurements (PRE, 20 Pitched, 40 Pitched, and 60 Pitched). We assumed a single-group design with a nonsphericity correction factor (ε) set to 1.0 and a moderate correlation between repeated measures (r = 0.5). As a reference for effect size, we used Mirabito et al. [6], who reported a large pre–post-pitching effect on IRROM (Cohen’s *d* = 0.917) in 10 collegiate baseball pitchers. This value was converted to an equivalent repeated-measures ANOVA effect size using the formula f = d/√2, which yielded f ≈ 0.648 under the assumption of r = 0.5. With α = 0.05, desired power (1 − β) = 0.80, four repeated measurements, and f = 0.648, the analysis indicated that a minimum of six participants would be required to detect a statistically significant within-subject effect. To improve statistical robustness and account for potential attrition, we recruited 15 participants.

### 2.2. Participants

Fifteen male collegiate baseball pitchers participated in this study (mean ± SD: age, 21.6 ± 2.4 years; height, 173.1 ± 3.5 cm; weight, 69.9 ± 6.4 kg; body mass index, 23.3 ± 2.3 kg/m^2^). All participants were active and capable of pitching with full effort with no pain or throwing-related symptoms, similar to the inclusion criteria described by Mirabito et al. [6]. None of the participants had a history of orthopedic surgery, fracture, or dislocation involving the shoulder or lower extremities. Participants with pain or functional limitations affecting their pitch ability were excluded. All pitchers had ≥5 years of competitive baseball experience (mean, 12.3 ± 1.9 years). The history of previous injuries was collected through self-report, and three participants reported previous shoulder injuries and two reported previous elbow injuries; however, none were symptomatic at the time of testing. All participants provided written informed consent before data collection. The Ethics Committee for Epidemiology of Hiroshima University approved this study (approval number: E2022-0185-01). All participants provided their informed written consent before participation, in accordance with the Declaration of Helsinki.

### 2.3. Procedures for Conducting Pitching Sessions and Measurement Sessions

All participants underwent a baseline measurement of shoulder ROM, foot morphology, intrinsic foot muscle properties, and GRF data before pitching (PRE). A pitching workload was played following the previous protocol [6]. The protocol comprised 60 pitches categorized into three sets of 20 pitches each, with each set being treated as a simulated “inning”. The ten-minute rest periods between sets were provided to mimic the in-game recovery intervals. After each set, the participants underwent repeated assessments of the ROM, foot morphology, and intrinsic muscle mechanical properties. During the pitching task, the participants drove from a flat surface to a catcher positioned 18.4 m away. The pitch types were randomly distributed throughout the sessions, comprising 36 fastballs, 12 curveballs, and 12 changeups, according to the pitch type distributions reported in the 2019 Major League Baseball season.

**Figure 1:**
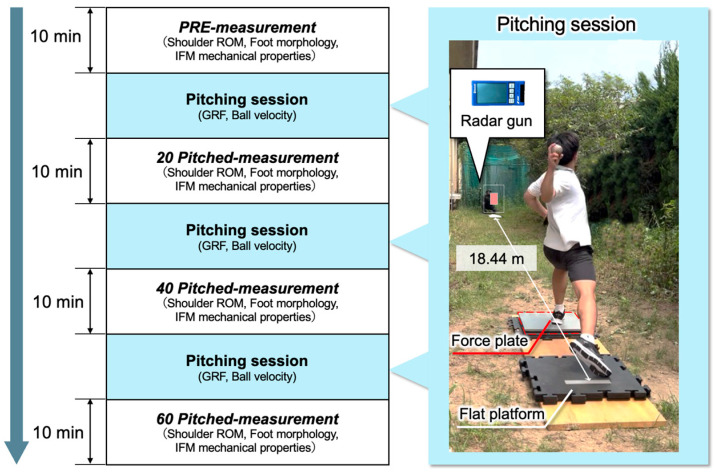
Experimental setup for the simulated pitching task.

In addition to the procedures described in a previous study [6], a force platform (AccuGait; AMTI, Watertown, MA, USA) was embedded in the stepping foot landing zone to collect GRF data. For each set, the GRF was recorded for the first three fastballs to analyze the loading response at the stride leg contact while maintaining fatigue and measurement conditions as consistent as possible. The velocity of the ball was recorded for the first three fastballs in each set using a radar gun (Multi–Speed Tester V BT; SSK Corp., Osaka, Japan) positioned behind the catcher. The mean value of three pitches was used as the representative pitch velocity for each set. The setup used for the pitching task is illustrated in Figure 1.

The participants pitched from a flat platform toward a catcher positioned 18.44 m away. A force plate was placed in the stride leg landing zone to record GRF data. The ball velocity was measured using a radar gun placed within 1.5 m behind the home plate. This setup was designed to replicate the actual game conditions while capturing biomechanical and performance-related data.

### 2.4. Measurement of ROM of the Shoulder Joint

The ROM of the shoulder joint was assessed for both internal and external rotations (measured in degrees) with the arm positioned at 90° of abduction. Measurements were performed on the dominant (throwing) arm. The participants were placed in the supine position on an examination table. A physical therapist stabilized the participant’s upper limb in the testing position while the assistant operated a goniometer (TTM–KO, SAKAI Medical Co., Tokyo, Japan). The movement axis was aligned with the forearm, and the reference axis was set parallel to the surface of the table [6]. The average of the three measurements was used in the analysis.

This measurement method demonstrated good reproducibility. To assess intra-rater reliability, ROM was measured at two time points on a separate day spaced 20 min apart. The reproducibility of the three repeated measurements at each time point was high, with an intraclass correlation coefficient (ICC) of (1,3) = 0.977. Additionally, the reproducibility between the two time points was good, with an ICC (3,1) of 0.896.

### 2.5. Morphological Assessment of the Foot

To quantify foot morphology, including the navicular height (mm), foot length (mm), and foot width (mm) of the stride leg, a three-dimensional foot scanner (INFOOT2 USB scanning system, IFU2–S–01, I–Ware Laboratory Co., Osaka, Japan) was used. This noncontact laser-scanning system utilizes eight synchronized cameras and four red-line lasers to capture the three-dimensional structure of the foot with high anatomical precision. The system has previously been validated for both inter- and intra-rater reliability and showed strong agreement with caliper-based and radiographic foot measurements [26,27]. The reliability of the INFOOT 3D scanner used in this study has been documented previously. Ballester et al. (2017) [28] reported excellent intra-rater reliability, with ICCs above 0.98 and SEM values generally <1 mm for linear foot measurements. Therefore, the INFOOT scanner was considered sufficiently reliable for use in the present analysis [28]. Before scanning, a trained technician with more than 5 years of experience placed skin markers on the following four anatomical landmarks of the stride leg: (1) the medial aspect of the first metatarsal head, (2) the lateral aspect of the fifth metatarsal head, (3) the most posterior aspect of the calcaneus, and (4) the navicular tuberosity. Participants were instructed to sit upright with their gaze forward, to ensure equal weight distribution across both feet. After scanning the foot in a non-weight-bearing seated position, a second scan was performed in a full weight-bearing standing posture. All measurements and 3D reconstructions were performed using the manufacturer’s software (Footprint Measurement, Measure; I-Ware Laboratory Co., Ltd., Osaka, Japan).

Leg–heel alignment (LHA) was measured using a method adapted from Maeda et al. [29] as rear foot posture with ImageJ 1.54 (National Institutes of Health, Bethesda, MD, USA). Participants were instructed to stand in a full weight-bearing double-limb position on a 30 cm high platform to allow proper visualization of the lower leg and rear foot. The LHA was defined as the angle between two bisecting lines: one line was drawn through the distal one-third of the lower leg, and the other line was drawn through the midline of the posterior aspect of the calcaneus. An LHA less than 0 indicates rearfoot eversion, whereas an LHA greater than 0 indicates rearfoot inversion. All measurements were taken from the posterior view while the participants kept a relaxed standing posture.

### 2.6. Mechanical Property Assessment of the Intrinsic Foot Muscle

A handheld myotonometer (MyotonPRO; Myoton AS, Tallinn, Estonia) was used to assess the mechanical properties of the AbH and PF in the stride leg. The stride leg is defined as the leg that steps forward toward the catcher during pitching. For example, in the case of a right-handed pitcher, the stride leg is the left leg. This device non-invasively quantifies the mechanical characteristics of soft tissues by applying a brief mechanical impulse and recording the resulting oscillatory response. The measured parameters included tone (Hz), stiffness (N/m), elasticity (log decrement), relaxation time (ms), and creep. Each of these metrics reflects distinct biomechanical properties of the tissue, including passive tension (tone), resistance to external deformation (stiffness), ability to return to its original shape after deformation (elasticity), time taken to recover the baseline state after force removal (relaxation time) and time-dependent deformation under sustained loading (creep) [30,31].

**Figure 2:**
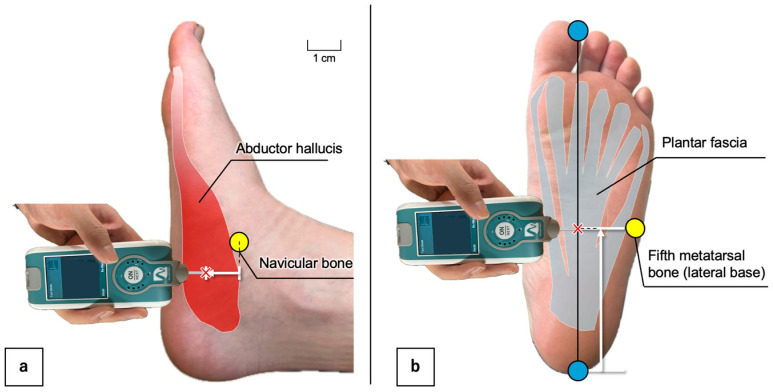
Measurement sites for assessing the mechanical properties of the abductor hallucis (**a**) and plantar fascia (**b**) using MyotonPRO.

All measurements were performed with the participants in the prone position, with the foot supported in neutral alignment and the muscles fully relaxed. The AbH was assessed in the muscle belly, located approximately 1 cm anterior to the navicular tuberosity, according to the electrode placement protocol described by Incel et al. [32] (Figure 2a). PF was measured on the central plantar surface of the foot at the level of the lateral base of the fifth metatarsal bone, as described by Orner et al. [31] (Figure 2b). A single examiner measured each site three times, and the average of the three trials was used for the analysis.

All measurements were conducted with the participants in a prone position, with the foot supported in a neutral position, and the muscles fully relaxed. Figure 2a; The AbH was assessed at the muscle belly approximately 1 cm inferoposterior to the navicular tuberosity, as described by Incel et al. [32]. Figure 2b; The PF was measured on the central plantar surface at the level of the lateral base of the fifth metatarsal bone, following the method of Orner et al. [31]. The test–retest reliability analyses demonstrated excellent repeatability for both muscles, with all intraclass correlation coefficient values exceeding 0.80 (Table A1) [33].

### 2.7. Measurement of the GRF of the Stride Leg During Pitching

A 49.5 × 49.5 cm force platform (AccuGait; AMTI, Watertown, MA, USA) was used to assess the GRF of the stride leg during straight pitching in repeated trials. The sampling frequency was set to 200 Hz, and the data were smoothed using a zero-lag second-order low-pass Butterworth filter with a cutoff frequency of 20 Hz. Before measurements, body weight (N) at rest was calculated as the average of the vertical GRF component during a 5 s quiet standing test on the force platform to normalize the GRF of the stride leg GRF to the body weight during pitching. For the analysis of GRF during repeated pitching, the point at which the vertical GRF first exceeded 5% of body weight was defined as foot contact (t = 0 s), and the GRF components within a 0.3 s window from this point were used for analysis. This analysis was based on GRF data collected from the first three straight pitches in each set. In this study, the peak GRF was identified within a 0.3 s window after SFC. The primary purpose of using this fixed window was to avoid including GRF generated by non-pitching actions (e.g., a jump or recovery step after ball release) that could exceed the forces produced during the pitching motion. The choice of the 0.3 s duration was based on previous reports indicating that shoulder torque returns close to zero within this timeframe [34]. During this phase, the stride leg plays a central role in receiving the GRF and transmitting energy to the upper limbs. Therefore, this interval was selected for the analysis. Within this interval, the peak values of the anterior–posterior, medial–lateral, and vertical GRF components were identified. These peak values were normalized to the body weight (N) and expressed as a percentage of body weight (%BW) to calculate the stride leg peak GRF-to-body weight ratio in each direction.

### 2.8. Data Analysis

Statistical analyses were performed with IBM SPSS Statistics for Windows, version 28.0 (IBM Corp., Armonk, NY, USA). The significance level was set at *p* < 0.05 for all analyses. The normality of each parameter was assessed using the Shapiro–Wilk test. To examine the time-dependent changes in each parameter between pitching sessions, repeated-measures analysis of variance (RM–ANOVA) was conducted. When a significant effect of time was observed, Bonferroni-adjusted pairwise comparisons were conducted. Effect sizes were calculated as generalized eta squared (η^2^G) using the spreadsheet provided by Lakens (2013) [35]. The change (Δ) between PRE and each pitch condition was calculated for IR, foot morphology, and muscle mechanical properties (Δ = value in each pitch set—value in PRE). The relationship between ΔIRROM and each Δ parameter was analyzed using Pearson’s correlation after confirming normality using the Shapiro–Wilk test. Correlation strength was interpreted according to Zieliński (2025) [36], with thresholds of *r* = 0.33 (small), *r* = 0.45 (medium), and *r* = 0.62 (large); correlations below 0.33 were regarded as clinically negligible [36]. For the GRF data, the raw values for each pitching condition were used instead of changing scores to preserve the assumed causal direction, where the GRF was considered a potential contributor to changes in IR and foot structure.

To address the potential risk of multicollinearity, covariates were selected based on their significant correlation with IR. When a pair of candidate covariates exhibited a high intervariate correlation (r ≥ 0.8), the variable with the strongest correlation to ΔIRROM was retained. This threshold was chosen based on previous literature, where correlation coefficients > 0.8 indicated problematic multicollinearity [37]. Details of this selection process are provided in Table A1. A linear mixed-effects model (LMM) was constructed with ΔIRROM as the dependent variable to identify factors associated with changes in IR. Fixed effects included the pitching condition (Time: 20 Pitched, 40 Pitched, and 60 Pitched) and the selected covariates. Random effects for participant ID were modeled to account for inter-individual variability. Finally, if a foot-related parameter was identified as a significant predictor in the LMM, its biomechanical relevance was further explored by examining its correlation with the GRF data using Pearson’s correlation analysis. Model assumptions were checked by inspecting the normality of residuals using Q–Q plots and the Shapiro–Wilk test, and by verifying homoscedasticity through residuals-versus-fitted plots. For all fixed-effect parameters estimated in the LMM, 95% confidence intervals (CIs) were calculated to indicate the precision of the estimates.

## 3. Results

### 3.1. Temporal Changes in Shoulder ROM, Foot Morphology, and Muscle Mechanical Properties During Repeated Pitching

**Figure A1:**
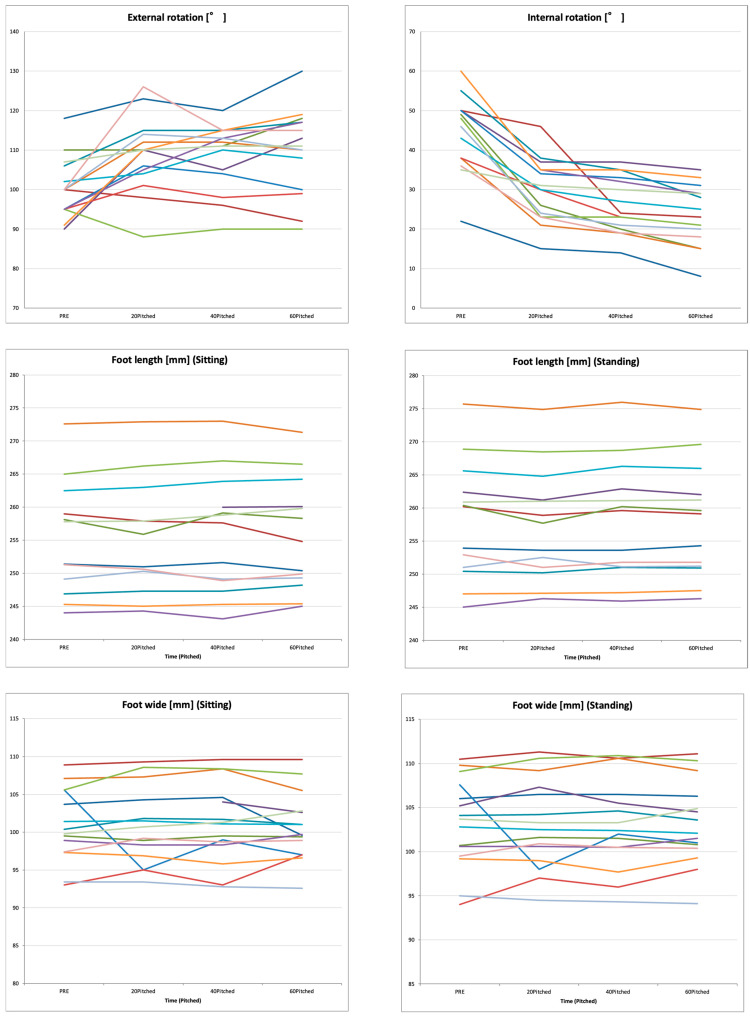

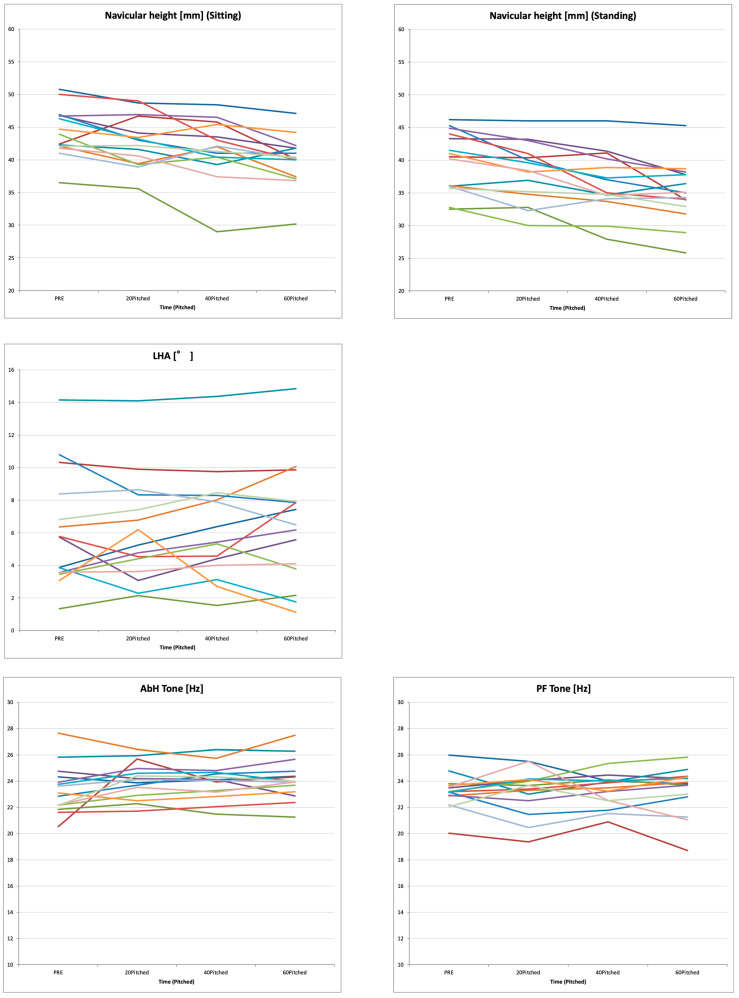

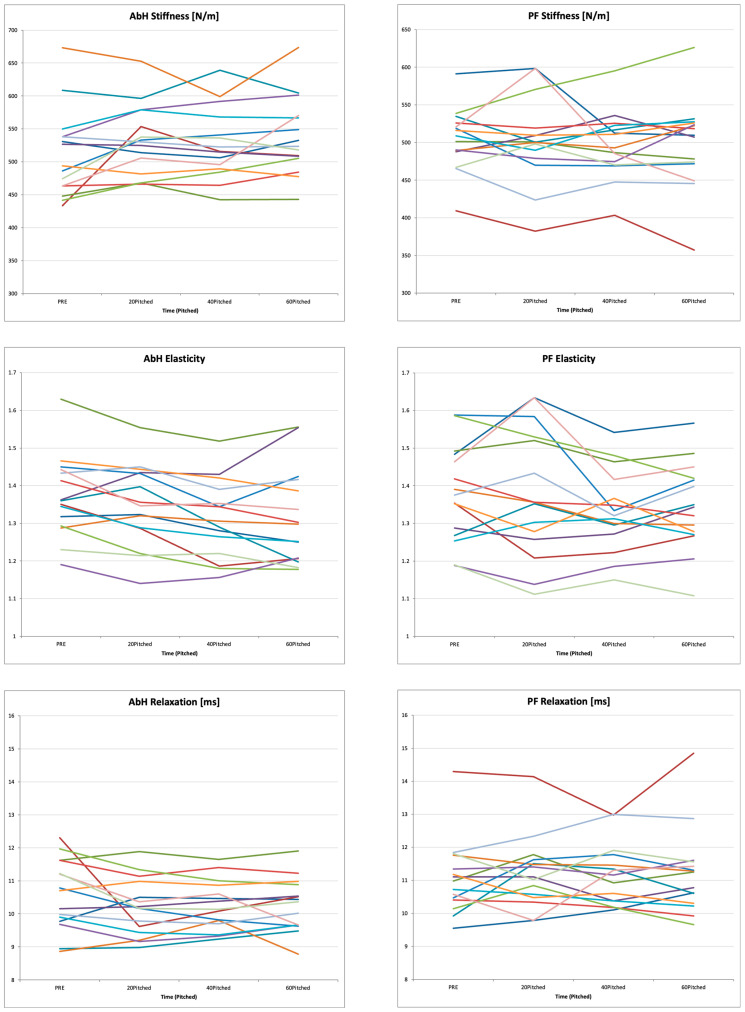

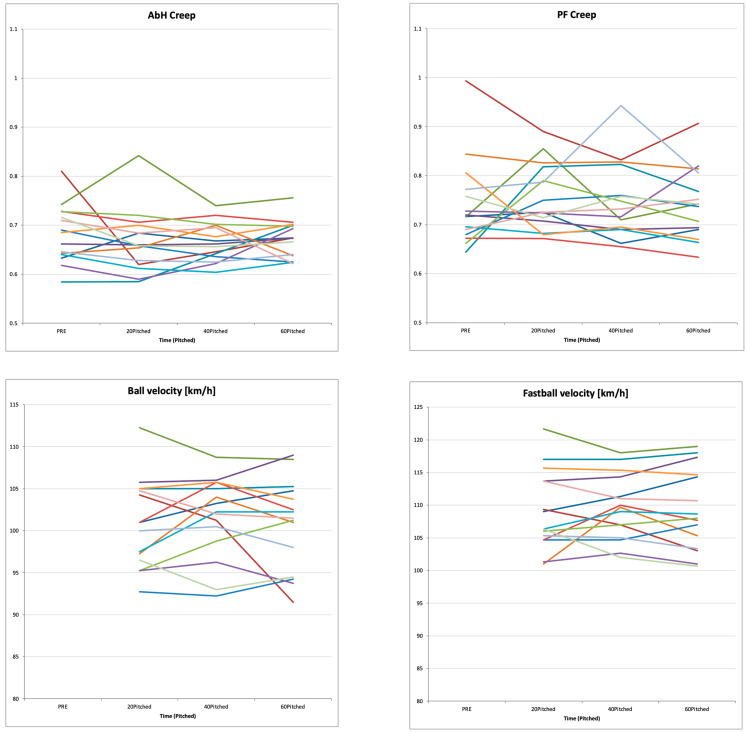

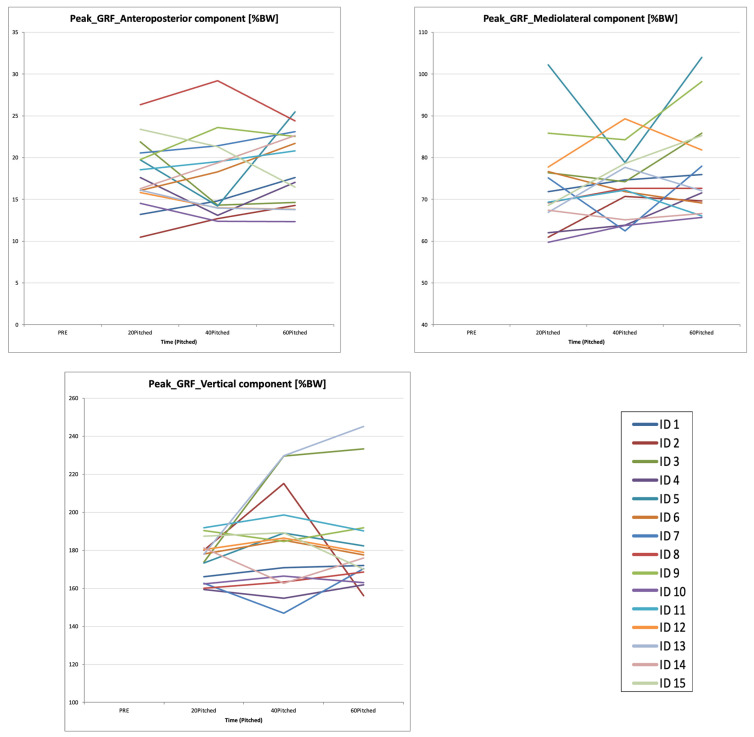
Individual trajectories of each variable across measurement sessions.

Table 1 lists the temporal changes in each parameter during repeated pitching. With an increasing number of pitches, the IRROM in the 90° abducted shoulder position decreased significantly (*p* < 0.01, η^2^G = 0.528), whereas the external rotation ROM increased significantly (*p* < 0.01, η^2^G = 0.162). Regarding foot morphology, the navicular height decreased significantly in both sitting and standing positions compared to PRE (sitting: *p* < 0.01, η^2^G = 0.141; standing: *p* < 0.01, η^2^G = 0.138). Regarding the mechanical properties of the muscle, the elasticity of the AbH muscle showed a significant reduction compared to PRE (*p* < 0.01, η^2^G = 0.048). In contrast, no significant changes were observed in the parameters related to the PF or GRF components associated with the pitching performance. Individual variation through time is shown in the Figure A1.

### 3.2. Correlations Between Changes in Shoulder IR and GRFs of the Lead Foot During Pitching

The mean change in shoulder internal rotation during pitching was −21.9° (95% CI, −26.1° to −17.8°). As presented in Table 2, changes in IRROM (ΔIRROM) were significantly correlated with several parameters related to foot morphology and the mechanical properties of the AbH. Specifically, significant positive correlations were observed between ΔIRROM and changes in navicular height, both in the sitting (r = 0.520, *p* < 0.01, medium) and standing positions (r = 0.596, *p* < 0.01, medium). Furthermore, the elasticity of the AbH showed a significant positive correlation with ΔIRROM (r = 0.427, *p* < 0.01, medium).

Conversely, a significant negative correlation was found between ΔIRROM and the vertical component of the GRF during lead foot contact in pitching (r = −0.380, *p* = 0.01, low).

### 3.3. Correlations Between Changes in Shoulder IR and GRFs of the Stride Leg During Pitching

Before performing the LMM analysis, potential covariates were identified based on the correlation analysis presented in Table 2. Variables that demonstrated a significant association with ΔIRROM and did not exhibit multicollinearity with one another (defined as r < 0.8) were selected as covariates. In cases where the correlation coefficient between two variables exceeded r = 0.8, the variable showing a stronger correlation with ΔIRROM was retained. As presented in the Table A2, because the changes in navicular height measured in sitting and standing positions were highly correlated, the standing measurement—which showed a stronger correlation with ΔIRROM—was selected as a covariate.

Table 3 presents the results of the LMM analysis. The model yielded a coefficient of determination of R^2^ = 0.596, indicating that approximately 59.6% of the variance in ΔIRROM was explained by the model. Regarding the time-related factors, ΔIRROM significantly increased after 20 (β = 3.494, *p* = 0.013) and 40 (β = 2.426, *p* < 0.01) pitches, compared with the reference condition of 60 pitches. Among the covariates, elasticity of the AbH showed a significant positive association with ΔIRROM (β = 29.235, *p* = 0.046). In contrast, the vertical component of the GRF was significantly and negatively associated with ΔIRROM (β = −0.087, *p* < 0.01), indicating that loading characteristics during pitching may influence changes in IRROM.

However, changes in navicular height in the standing position (ΔStand_Navicular_Height) showed no statistically significant association with ΔIRROM (β = 0.465, *p* = 0.192).

### 3.4. Relationship Between GRF Components and Changes in Muscle Elasticity and Pitching Performance

Table 4 presents the results of the LMMs examining the relationships among GRF components, AbH elasticity, and fastball velocity. Among the GRF components, the vertical component was significantly negatively associated with changes in AbH elasticity (β = −0.001, *p* = 0.047), suggesting that increased vertical loading during pitching reduces the elasticity of the AbH muscle. In contrast, the anteroposterior GRF component was significantly and positively associated with fastball velocity (β = 0.363, *p* = 0.038), indicating that greater anteroposterior loading may contribute to improved pitching performance. No significant associations were observed between the mediolateral and vertical GRF components and any of the variables.

## 4. Discussion

To the best of our knowledge, this is the first study to investigate temporal changes in shoulder IRROM associated with repetitive pitching while simultaneously considering alterations in foot morphology, muscle mechanical properties, and GRFs using an LMM. Functional changes in the upper extremities. Furthermore, changes in the elasticity of the AbH and vertical GRF were significantly associated with ΔIRROM, suggesting that alterations in the structure of the lower extremities and loading mechanics can contribute to functional changes in the upper extremities. These findings provide novel insights into integrated biomechanical adaptations during repetitive pitching and may inform preventive strategies for reducing shoulder motion deficits in baseball players.

A previous study evaluating college baseball pitchers reported a decrease in the IRROM of approximately 18° after one game [38], whereas another study involving professional players found a reduction of approximately 10° [39]. These findings indicate that acute reductions in IRROM occur regardless of competitive level. Similarly, in this study, we observed an average decrease of approximately 20° in the IRROM between pitching sessions. The underlying mechanisms of this phenomenon were explored in detail using a cadaveric model of the throwing shoulder [40]. Their findings suggested that repeated overhead motion led to anterior laxity of the glenohumeral joint capsule, predisposing the humeral head to shift anteriorly. Owing to the limited volumetric capacity of the capsule, this anterior displacement resulted in a relative restriction of posterior flexibility, thereby limiting the IR. Additionally, Mirabito et al. [6] reported that changes in the morphology and elasticity of the rotator cuff muscles, such as the infraspinatus and teres minor muscles, may also contribute to the immediate post-pitching reduction in IRROM. Mirabito et al. did not specify the mechanism underlying this result [6]; however, they speculated that the immediate change may have been caused by the eccentric contraction of the posterior rotator cuff muscles, such as the infraspinatus and teres minor, which draw the humeral head toward the glenoid in response to the traction force of up to 1000 N acting on the shoulder joint at the time of ball release. Overall, these studies suggest that changes in humeral head positioning, uneven capsular tension, and altered mechanical properties of the rotator cuff work synergistically to reduce the IR after pitching [40]. Although this IR reduction typically persists for more than 24 h and tends to return to baseline within several days [6,39], continued high-frequency pitching may lead to repetitive posterior–superior translation of the humeral head during external rotation, which in turn could increase the risk of posterior–superior labral injuries and internal impingement [4,39].

In this study, a gradual decrease in navicular height was observed on the stride during repeated pitching. This transient change may reflect an acute increase in the flexibility of the tendon structures that support the MLA owing to repetitive loading. Repeated foot strikes during the stride likely induced a dynamic stretch effect on the AbH and PF, which are the key components of the MLA. A previous study [41] demonstrated that dynamic stretching acutely increased muscle flexibility and joint ROM, particularly during short bouts of repetitive activity. Consequently, the observed reduction in navicular height may be attributable to increased tissue extensibility caused by repeated loading-induced stretching of the soft foot tissues. Additionally, as the number of pitching sets increased, both the stiffness and elasticity of the AbH increased significantly, whereas no notable changes were observed in static muscle tone, relaxation, and creep. Previous studies have demonstrated that eccentric contractions improve passive tension by increasing the stiffness of titin, a key structural protein within the muscle tissue [42,43]. In the context of pitching, dynamic lowering of the navicular bone transiently flattens the MLA, which likely leads to repeated eccentric contractions of the AbH as it resists this downward motion. Eccentric contractions under such mechanical loading are known to induce microdamage and localized fluid accumulation in the muscle, resulting in increased intramuscular pressure [44]. Increased intramuscular pressure raises the muscle’s internal resistance to deformation and reduces energy loss during recoil, reflecting increased stiffness and lower damping. This pressure–elasticity relationship has been demonstrated in human studies; applying blood flow restriction to lower leg muscles to elevate pressure has been shown to increase muscle elasticity [45]. In this study, an increase in AbH stiffness was also observed. According to a review on exercise-induced changes in muscle stiffness, this phenomenon has been most frequently reported in studies employing eccentric training protocols performed at intensities above 70% of one repetition maximum with more than 40 repetitions. This finding is consistent with the present study, in which an increase in stiffness was observed after more than 40 pitches. The review further suggested that these stiffness changes are likely attributable to a disruption of intracellular calcium homeostasis [46]. Alternatively, considering that the AbH is one of the muscles supporting the MLA, it is notable that the present study observed a decrease in navicular height even in the seated position as the pitching sets progressed. Kobayashi et al. [25] reported that flattening of the MLA may elongate the AbH and increase its stiffness. Therefore, the progressive decrease in navicular height during the pitching protocol may have caused the AbH to become increasingly stretched, like a bowstring, leading to elevated stiffness. In summary, the observed increases in AbH stiffness and elasticity during repeated pitching can be interpreted as the combined result of physiological factors such as elevated intramuscular pressure and physical factors related to MLA structural deformation. However, this study cannot determine the causal relationship between AbH and MLA structural changes, and the mechanical properties of other intrinsic foot muscles besides the AbH and PF were not evaluated; therefore, further investigation including these factors is warranted. However, in our study, no significant changes were observed in PF properties. Several factors may explain why PF mechanical properties did not significantly change during repeated pitching. First, the magnitude of impact loading during pitching is generally lower than that during running. Running generates a vertical GRF of approximately 250% BW per step [47], whereas the vertical GRF during pitching in this study was approximately 220% BW. Second, in baseball, the stride foot typically lands with a rearfoot strike pattern. A previous study in runners reported that those who used a rearfoot strike exhibited smaller post-run reductions in PF stiffness than forefoot strikers, suggesting that the rearfoot strike pattern during the pitching stride may help maintain PF stiffness [48]. Finally, pitching is a discontinuous, ballistic motion, unlike the cyclic nature of running, with brief rest periods between throws (e.g., while the catcher returns the ball). PF stiffness has been shown to recover rapidly during rest after high-intensity running [49], and this difference in task periodicity may also contribute to the absence of PF mechanical changes observed in this study. Thus, it is plausible that the moderate cumulative load during pitching was sufficient to induce acute changes in the mechanical properties of AbH, but not in PF. These findings suggest that 60 consecutive pitches impose a mechanical load capable of producing an immediate reduction in navicular height of the stride leg and an increase in AbH elasticity.

In the correlation analysis presented in Table 2, greater increases in seated and standing navicular height and AbH elasticity, as well as lower vertical GRF, were associated with more pronounced reductions in IR. However, the LMM results in Table 3 show that only AbH elasticity and vertical GRF were significant predictors of IR, with navicular height showing no significant associations. This discrepancy suggests that the dynamic behaviors of the foot and intrinsic muscles during pitching may have a stronger association with shoulder mobility than static foot morphology. Based on these LMM results, we propose two hypothetical mechanisms. First, from the perspective that the mechanical properties of the AbH may have influenced ΔIRROM, AbH elasticity, as measured using MyotonPRO, reflects the damping of muscle oscillations; higher values indicate faster recovery from deformation, specifically greater “bounciness” [50]. Therefore, individuals with increased ΔAbH elasticity are likely to exhibit faster rebound of the MLA during foot loading, contributing to greater restoration of navicular height. This is consistent with previous findings showing that variability in navicular height during gait is associated with AbH morphology [51]. The high dynamic rebound of the AbH may inhibit the deflection of the MLA, and as a result, the vertical GRF generated at SFC may not be attenuated, potentially increasing the actual kinetic energy transmitted to the upper segments, including the trunk and upper limb. Indeed, vertical GRF was significantly correlated with ΔIRROM in this study. A previous study reported that a higher vertical GRF increases the stride leg hip adduction moment, which augments shoulder external rotation torque [52]. This interpretation aligns with the kinetic-chain framework described by Kibler and Sciascia [8,12], who emphasized that impairments in force transfer or segmental sequencing at any link of the chain can influence glenohumeral motion and loading demands. Repetition of this kinetic sequence may exacerbate shoulder rotation and promote anterior humeral translation, thereby contributing to GIRD. A second hypothetical mechanism is that the decrease in ΔIRROM is primarily driven by higher vertical GRF, and that the change in AbH elasticity is one of the secondary responses to this loading pattern. A higher vertical GRF tends to flatten and pronate the MLA, requiring the AbH to repetitively lengthen and shorten to support the arch from the plantar–medial side. As a result, AbH elasticity may appear altered in participants who experienced greater vertical GRF, thereby showing an association with ΔIRROM. This explanation may be superior in that it does not treat GRF as the outcome of a single small foot muscle but as the product of the whole foot–lower-limb kinetic system. Previous studies that linked foot morphology and function to throwing-related injury risk have reported a higher risk in athletes with a flattened MLA or impaired toe function, and, when interpreted together with the present findings, these foot characteristics may represent a state in which long-term repetition of excessive GRF has already led to structural deterioration of the foot [18,53]. Because reductions in foot structure and function may not only increase the risk of throwing-related disorders but also predispose athletes to future lower-limb or trunk musculoskeletal conditions, further studies are needed to clarify the causal relationships underlying these mechanisms.

These findings suggest that increased AbH elasticity may be associated with fluctuations in the vertical GRF and shoulder range of motion of the athlete, and describe foundational information leading to the assessment and care of the baseball pitcher’s post-pitching foot.

Clinicians treating throwing-related shoulder injuries have long used rotator cuff training, full-body stretching, and various other strategies to maintain pitch performance. If the results of the present study and hypothetical mechanisms are substantiated, adding to them a reduction in the vertical component of GRF and inhibition of structural changes in the foot may help to maintain the kinetic chain of the lower limb and ultimately further reduce the accumulation of mechanical stress on the shoulder joint. From a neuromechanical pathways perspective, increased medial arch flexibility may reduce proprioceptive input and impair lower-limb coordination. Because pitching relies on efficient ground-up force transmission, diminished arch control in the stride leg could disrupt segmental timing and contribute to greater shoulder loading or acute IRROM loss [54]. Clinicians should evaluate arch height and flexibility when managing pitchers, and incorporate foot-core, trunk-stability, and proprioceptive exercises to enhance lower-limb stability and maintain kinetic-chain efficiency.

Furthermore, as presented in Table 4, the vertical GRF does not appear to contribute to fastball velocity, whereas the horizontal components (anterior–posterior and medial–lateral) are likely to play a greater role. This aligns with the findings of a previous study by Wasserberger et al. [55]. Therefore, minimizing the vertical GRF can alleviate the mechanical stress on the AbH, help stabilize dynamic changes in navicular height, and allow pitchers to sustain their performance over longer innings. To achieve this, it is important to adopt a pitching form where the stride-leg knee is maintained in extension, thereby limiting excessive translational pelvic motion in the direction of the throw while the feet are in contact with the ground. Additionally, improving AbH endurance through exercises such as short-foot training, rock–scissors–paper movements, and towel-gathering may serve as an effective strategy to minimize breakdown of the MLA. However, these interventions may not provide immediate effects and could be less practical for amateur pitchers with limited practice time. In such cases, simpler approaches, including soft taping of the AbH to support the MLA or the use of heel pads and other foot-cushioning materials, may be practical alternatives. In addition, physical therapists and athletic trainers should also consider adopting reliable foot-posture assessments for monitoring foot conditions resulting from pitching. The arch height index provides an objective measurement of the morphology of the MLA [56], and the FPI-6 not only distinguishes a pronated foot using a cutoff score of 6 but may also be able to identify clinically meaningful deviations in foot posture (i.e., changes of two points or more) [57]. By using both assessments together, clinicians can more accurately capture deformation of the MLA and the associated compensations in the rearfoot and forefoot. These approaches can reduce the loss of shoulder IR range during repetitive pitching without compromising pitching performance.

This study has several limitations. First, the present study was designed as a descriptive observational study and therefore cannot establish causal relationships among changes in IRROM, MLA morphology, AbH and PF mechanical properties, and GRF. In addition, the acute responses of the shoulder muscles were not evaluated; therefore, changes in the morphology of the infraspinatus and teres minor after pitching and their contribution to IRROM loss were only indirectly inferred. Similarly, the mechanical properties of the AbH and PF were assessed under unloaded, passive conditions, which may not reflect their functional activities such as muscle activation under the high GRF generated during pitching. Second, the experimental environment differed from actual game conditions. Because a rigid force platform was used and cleats could not be worn, the impact on the foot might have been greater than on natural ground, possibly leading to an overestimation of the changes in navicular height. Third, GRF, trunk rotation, knee extension, and other kinetic-chain parameters were not synchronously processed, making it impossible to analyze their temporal relationships. Moreover, only the first three fastballs of each set were analyzed, which may have overlooked subsequent biomechanical changes. Fourth, the absence of a 24 h follow-up assessment limits the ability to determine whether IRROM, MLA height, and tissue mechanical properties returned to baseline or persisted beyond the pitching session. Finally, the sample consisted of pitchers with specific performance levels, limiting the generalizability of the findings to players at different developmental stages. Although vertical GRF was not significantly associated with ball velocity, a positive trend was observed, suggesting that a larger sample might reveal a significant contribution of vertical loading to pitching performance.

## 5. Conclusions

This study suggests that the reduction in shoulder IR observed during repetitive pitching may be accompanied by concurrent changes in foot muscle elasticity and vertical GRFs in the stride leg. Specifically, increased AbH elasticity and elevated vertical GRF were associated with a greater decrease in the IRROM of the shoulder. These findings indicate a potential interrelationship between the dynamic responses of the foot—particularly the behavior of the MLA—and upper-limb motion through the kinetic chain. Because vertical GRF is unrelated to ball speed, strategies to suppress it may help maintain shoulder joint ROM without affecting performance. From a clinical perspective, athletic trainers and physical therapists should pay attention not only to shoulder flexibility but also to stride-leg stability and foot intrinsic muscle condition during pitching. Simple interventions such as intrinsic foot muscle training or foot cushioning, and monitoring foot posture and MLA-supporting intrinsic muscles during the season may help reduce excessive vertical loading and preserve shoulder mobility. However, as this study involved a relatively small and homogeneous group of pitchers, the findings should be interpreted with caution. Future studies including a larger and more diverse cohort of athletes with varying performance levels are needed to confirm these associations and further clarify the long-term implications of these biomechanical interactions. Given its limited sample size and exploratory design, this research should be regarded as a pilot study intended to generate hypotheses for future investigations.

## Figures and Tables

**Table 1:** Changes in each parameter over time during repetitive pitching.

Parameter	PRE	20 Pitched	40 Pitched	60 Pitched	F	*p*	η^2^G
*1. Shoulder ROM in 90°-Abducted*							
External rotation [°]	100.3 ± 7.5	108.8 ± 9.4 *	108.5 ± 8.31 **	109.9 ± 10.8 *	11.048	*<0.001*	0.162
Internal rotation [°]	45.3 ± 10.2	29.9 ± 8.0 *	26.1 ± 7.12 **	23.4 ± 7.6 **^,††,§§^	76.262	*<0.001*	0.528
*2. Foot morphological parameter*							
Foot length [mm] (Sitting)	255.3 ± 8.1	255.2 ± 8.8	255.7 ± 9.0	255.6 ± 8.4	0.090	0.965	0.074 × 10^−3^
Foot length [mm] (Standing)	257.9 ± 8.6	257.5 ± 8.5	258.1 ± 9.0	258.0 ± 8.7	0.085	0.956	0.078 × 10^−2^
Foot wide [mm] (Sitting)	101.0 ± 4.7	100.7 ± 5.1	101.1 ± 5.2	100.7 ± 4.4	0.079	0.903	0.052 × 10^−2^
Foot wide [mm] (Standing)	103.2 ± 5.1	103.1 ± 5.1	103.1 ± 5.1	103.1 ± 4.7	0.012	0.998	0.042 × 10^−3^
Navicular height [mm] (Sitting)	44.3 ± 3.7	42.8 ± 3.8	41.7 ± 4.6 *	40.0 ± 3.8 **^,††^	13.715	*<0.001*	0.141
Navicular height [mm] (Standing)	39.7 ± 4.6	38.1 ± 4.5 *	36.4 ± 4.6 **	35.1 ± 4.5 **^,††^	19.836	*<0.001*	0.138
LHA [°]	6.1 ± 3.5	6.1 ± 3.2	6.3 ± 3.3	6.5 ± 3.6	0.364	0.707	0.002
*3. Mechanical properties of muscle*							
*3-1. Abductor Hallucis*							
Tone [Hz]	23.4 ± 1.8	24.1 ± 1.4	24.0 ± 1.3	24.1 ± 1.5	3.137	0.067	0.043
Stiffness [N/m]	511.2 ± 66.0	532.7 ± 53.3	527.5 ± 53.4	537.8 ± 58.5 *	3.450	*0.025*	0.031
Elasticity	1.37 ± 0.11	1.35 ± 0.11	1.31 ± 0.10 **^,†^	1.32 ± 0.13 *	6.218	*0.007*	0.048
Relaxation Time [ms]	10.6 ± 1.1	10.2 ± 0.9	10.3 ± 0.7	10.2 ± 0.8	2.364	0.123	0.023
Creep	0.70 ± 0.10	0.67 ± 0.06	0.67 ± 0.04	0.67 ± 0.04	0.611	0.574	0.014
*3-2. Planter Fascia*							
Tone [Hz]	23.2 ± 1.3	23.2 ± 1.7	23.3 ± 1.21	23.3 ± 1.8	0.053	0.984	0.001
Stiffness [N/m]	504.4 ± 40.9	503.3 ± 57.3	496.6 ± 43.9	497.9 ± 58.4	0.281	0.740	0.005
Elasticity	1.40 ± 0.10	1.38 ± 0.17	1.33 ± 0.11	1.34 ± 0.12	2.567	0.067	0.025
Relaxation Time [ms]	11.1 ± 1.1	11.2 ± 1.1	11.2 ± 0.9	11.2 ± 1.3	0.272	0.845	0.003
Creep	0.74 ± 0.10	0.76 ± 0.07	0.75 ± 0.08	0.74 ± 0.07	0.321	0.746	0.006
*4. Pitching performance*							
Ball velocity [km/h]		100.9 ± 5.3	101.7 ± 4.8	100.8 ± 5.4	0.475	0.627	0.006
Fastball velocity [km/h]		109.0 ± 6.0	109.6 ± 5.0	109.2 ± 6.2	0.243	0.742	0.002
*5. Pitching kinetics parameter*							
Peak_GRF_Anteroposterior component [%BW]		18.0 ± 4.1	17.5 ± 4.9	18.7 ± 4.4	0.742	0.476	0.013
Peak_GRF_Mediolateral component [%BW]		72.7 ± 10.8	73.3 ± 7.7	77.5 ± 11.6	2.926	0.082	0.045
Peak_GRF_Vertical component [%BW]		175.0 ± 10.9	184.9 ± 25.4	182.5 ± 25.1	1.662	0.210	0.039

Data are expressed as mean ± SD. Italic *p* values indicate statistically significant differences between the times (*p* < 0.05). *: compared with PRE (*p* < 0.05), **: compared with PRE (*p* < 0.01), †: compared with 20 Pitched (*p* < 0.05), ^††^: compared with 20 Pitched (*p* < 0.01), ^§§^: compared with 40 Pitched (*p* < 0.01). *p* values were adjusted using the Bonferroni correction for multiple comparisons. Generalized η^2^: Gη^2^; ROM: range of motion; GRF: ground reaction force; LHA: leg–heel alignment.

**Table 2:** Correlations between change in IRROM and temporal changes in foot structure, mechanical properties, and muscle morphology.

	ΔIRROM [°]		
	r	*p*	95% CI
Δ Navicular height (sitting) [mm]	0.520 **	<0.001	[0.27, 0.71]
Δ Navicular height (standing) [mm]	0.596 **	<0.001	[0.38, 0.76]
Δ Leg heel angle [°]	0.141	0.299	[−0.17, 0.42]
Δ AbH Tone [Hz]	−0.118	0.368	[−0.40, 0.19]
Δ AbH Stiffness [N/m]	−0.151	0.250	[−0.43, 0.16]
Δ AbH Elasticity	0.427 **	<0.001	[0.13, 0.65]
Δ AbH Relaxation [ms]	0.083	0.527	[−0.23, 0.38]
Δ AbH Creep	−0.053	0.689	[−0.35, 0.26]
Δ PF Tone [Hz]	−0.049	0.713	[−0.34, 0.27]
Δ PF Stiffness [N/m]	0.015	0.908	[−0.28, 0.31]
Δ PF Elasticity	0.181	0.167	[−0.13, 0.46]
Δ PF Relaxation [ms]	−0.117	0.374	[−0.40, 0.19]
Δ PF Creep	−0.146	0.267	[−0.43, 0.16]
Fastball velocity [km/h]	0.013	0.933	[−0.28, 0.30]
Peak_GRF_Anteroposterior component [%BW]	0.189	0.213	[−0.12, 0.47]
Peak_GRF_Mediolateral component [%BW]	0.234	0.122	[−0.08, 0.50]
Peak_GRF_Vertical component [%BW]	−0.380 *	0.010	[−0.61, −0.10]

Δ means the change value between each pitching session and PRE, IRROM: shoulder internal rotation range of motion; AbH: abductor hallucis; PF: plantar fascia, GRF: Ground reaction force; BW: body weight. All correlations were computed using Pearson’s method (*n* = 45, df = 43). 95% confidence intervals (CI) were estimated by Fisher’s z-transformation. Significant correlations are marked with * for *p* < 0.05 and ** for *p* < 0.01.

**Table 3:** Evaluation of temporal changes in foot morphology, pitching kinetics, and mechanical properties associated with shoulder internal rotation using a Linear Mixed-effects Model.

R^2^	Fixed Effect	Estimate (β)	SE	Df	t-Value	*p*-Value	95% CI
0.596	Intercept	−2.303	7.653	35.109	−0.301	0.765	[−17.838, 13.233]
	Time = 20 Pitched	3.494	1.920	34.278	1.820	0.077	[−0.406, 7.395]
	Time = 40 Pitched	2.426	1.067	26.707	2.274	0.031 *	[0.236, 4.616]
	Time = 60 Pitched	Reference	-	-	-	-	-
	ΔStand_Navicular_Height	0.465	0.350	35.945	1.330	0.192	[−0.244, 1.174]
	ΔAbH_Elasticity	29.235	14.164	38.095	2.064	0.046 *	[0.978, 55.257]
	Peak_GRF_Vertical_component	−0.087	0.036	30.666	−2.424	0.021 *	[−0.160, −0.014]

Δ means the change value between each pitching session and PRE; AbH: abductor hallucis; GRF: ground reaction force; SE: Standard Error; df: Degrees of Freedom; CI: Confidence Interval; Significant correlations are marked with * for *p* < 0.05.

**Table 4:** Correlation between ground reaction force components and changes in abductor hallucis elasticity and fastball velocity using Linear Mixed-effects Models.

Fixed Effect	Estimate (β)	SE	Df	t-Value	*p*-Value	95% CI
*ΔAbH_Elasticity*						
Intercept	0.094	0.094	43.626	0.993	0.326	[−0.096, 0.283]
Peak_GRF_Anteroposterior component	−0.003	0.003	44.917	−1.240	0.221	[−0.008, 0.002]
Peak_GRF_Mediolateral component	0.001	0.001	43.699	1.007	0.320	[−0.001, 0.002]
Peak_GRF_Vertical component	−0.001	0	43.258	−2.050	0.047 *	[−0.002, −0.0001]
*Fastball velocity [km/h]*						
Intercept	99.878	6.036	32.118	16.546	<0.001	[87.584, 112.172]
Peak_GRF_Anteroposterior component	0.363	0.167	31.795	2.170	0.038 *	[0.022, 0.703]
Peak_GRF_Mediolateral component	−0.043	0.051	33.479	−0.839	0.407	[−0.148, 0.061]
Peak_GRF_Vertical component	0.032	0.028	29.837	1.148	0.260	[−0.025, 0.088]

Δ means the change value between each pitching session and PRE; AbH: abductor hallucis; GRF: Ground reaction force, BW: body weight. Significant correlations are marked with * for *p* < 0.05.

## Data Availability

The datasets used and/or analyzed in the current study are available from the corresponding author upon reasonable request.

## Abbreviations

The following abbreviations are used in this manuscript:

AbH	Abductor hallucis
BW	Body weight
CI	Confidence interval
GIRD	Glenohumeral internal rotation deficit
GRF	Ground reaction force
ICC	Intraclass correlation coefficient
IR	Internal rotation
LHA	Leg–heel alignment
LMM	Linear mixed-effects model
MLA	Medial longitudinal arch
PF	Plantar fascia
ROM	Range of motion

## Appendix A

**Table A1:** Reliability metrics of muscle mechanical properties measured using the MyotonPRO device.

Muscle	Property	ICC (1,1)	SEM	MDC95
AbH	Tone [Hz]	0.90	0.6	1.6
	Stiffness [N/m]	0.98	10.1	28
	Elasticity	0.92	0.036	0.099
	Relaxation [ms]	0.96	0.2	0.6
	Creep	0.95	0.013	0.036
PF	Tone [Hz]	0.88	0.3	1.0
	Stiffness [N/m]	0.91	12.4	34.4
	Elasticity	0.96	0.028	0.078
	Relaxation [ms]	0.89	0.2	0.7
	Creep	0.94	0.015	0.041

AbH, abductor hallucis; PF, plantar fascia; ICC, intraclass correlation coefficient; SEM, standard error of measurement; MDC95, minimal detectable change at the 95% confidence level.

**Table A2:** Correlation matrix among candidate covariates considered during the variable selection process for the LMM.

	Δ Navicular Height (Sitting) [°]		Δ Navicular Height (Standing) [°]		Δ AbH Elasticity	
	r	*p*	r	*p*	r	*p*
Δ Navicular height (sitting) [mm]	-	-	-	-	-	-
Δ Navicular height (standing) [mm]	0.856 **	<0.001	-	-	-	-
Δ AbH Elasticity	0.233	0.073	0.274 *	0.034	-	-

ΔIRROM; change in shoulder internal rotation; AbH: abductor hallucis; PF: plantar fascia. Significant correlations are marked with * for *p* < 0.05 and ** for *p* < 0.01.
